# PLOD3 Variably Regulates Diverse Phenotypes in Human Cancer Cells

**DOI:** 10.3390/ijms27209336

**Published:** 2026-10-21

**Authors:** Xiaoxiao Zhang, Mingmin Zhang, Xue Zhang, Kexin Chen, Yilian Wang, Yan Zeng

**Affiliations:** Department of Zoology, College of Life Sciences, Nanjing Agricultural University, Nanjing 210095, China

**Keywords:** PLOD3, cancer, apoptosis, mitochondrial metabolism

## Abstract

PLOD3 (procollagen-lysine, 2-oxoglutarate 5-dioxygenase 3) is an essential enzyme that modifies collagen and contributes to collagen assembly and function. PLOD3 expression has been linked to human cancers and patient prognosis, although how PLOD3 regulates tumorigenesis is poorly understood. In this study we first examined the association between PLOD3 expression and human cancer survival and found that, in contrast to previous reports, PLOD3 expression was not significantly correlated with overall survival in cancers. We then studied how decreasing and increasing PLOD3 levels affected the phenotypes of HeLa, HT-1080, and HCT116 cells, three diverse and representative human cancer cell lines. Altering PLOD3 expression elicited a variable effect on the cell cycle and cell migration in the three cell lines. However, increased PLOD3 induced apoptosis, elevated the mitochondrial membrane potential, and reduced the reactive oxygen species (ROS) levels in all three cell lines, whereas decreasing PLOD3 had the opposite effects. Subsequent global mRNA expression analyses revealed changes in biological processes and pathways that were consistent with these phenotypic changes and suggested that apoptosis and mitochondrial metabolism might be two important mechanisms by which PLOD3 regulates cell behaviors.

## 1. Introduction

PLOD3 is one of the three enzymes (E.C. 1.14.11.4, along with PLOD1 and PLOD2) that hydroxylate lysine residues to produce hydroxylysines in collagen, and it further performs collagen O-linked glycosylation [1,2]. These modifications are necessary for the biosynthesis, assembly, and functions of collagen. Collagen is the most abundant protein in humans and a major component of the extracellular matrix (ECM), which is essential for the structural integrity and functions of tissues and impacts normal development and diseases such as fibrosis and cancer [3,4,5]. Not surprisingly, therefore, the complete loss of PLOD3 causes embryonic lethality in mice [6,7], and a number of human genetic connective tissue disorders harbor PLOD3 mutations [8,9,10,11,12,13].

Owing to the link between ECM and tumorigenesis, PLOD3 has been examined for its potential involvement in human cancers. The expression of PLOD3, mostly examined at the mRNA level, is elevated in gastric cancer [14,15,16,17], colon cancer [18,19,20,21], liver cancer [22,23,24], glioma [25,26], lung cancer [27,28,29,30], renal cell carcinoma [31,32,33,34], ovarian cancer [35], pancreatic cancer [36], bladder cancer [37], head and neck cancer [38], and cervical cancer [39]. In addition, Kaplan–Meier (KM) plotting revealed a positive correlation between high PLOD3 expression and poor prognosis for lung cancer [26,27,28,29], glioma [25,26], gastric cancer [16,17], liver cancer [23,24], renal cell carcinoma [32,33,34], colon cancer [19,20,21,40], bladder cancer [37], head and neck cancer [38], and cervical cancer [39]. In terms of the mechanisms by which PLOD3 promotes carcinogenesis, PLOD3 might regulate lung cancers via the STAT3 and PKC-delta signaling pathways [27,29,41], induce TWIST1 expression or the NEDD4/GOT2 axis to facilitate renal cell carcinoma [31,34], and activate T cells via the TNF-α/NF-κB pathway or induce TM9SF4-mediated autophagy in colon cancer [21,40]. There is little overlap in the reported PLOD3 mechanisms in distinct cancers, suggesting that the regulation by PLOD3 is complex, but it may also indicate that our mechanistic understanding is seriously lagging and fragmented. This is because previous work had focused on one or a few cell lines from the same cancers, and different researchers often employed different experimental assays, which prevents side-by-side comparisons and systemic analyses of PLOD3’s roles in human cancer cells.

In the current study we used three diverse human cancer cell lines: HeLa, HT-1080, and HCT116, as models to study PLOD3 functions. HeLa is a human cervical cancer cell line, HT-1080 is a human fibrosarcoma cell line, and HCT116 is a human colon cancer cell line. These cell lines are the popular representatives in the studies of their respective cancers, and PLOD3 has been investigated in the context of colon cancer [18,19,20,21] and cervical cancer [39], while PLOD3 expression is higher in sarcoma than in the adjacent normal tissue [26]. We investigated the effects of RNA interference (RNAi)-mediated reduction in PLOD3 as well as PLOD3 overexpression on the cell cycle, apoptosis, cell migration, and metabolism in the three cell lines. We then performed high-throughput RNA sequencing (RNA-seq) experiments and data analyses to examine gene expression and pathway changes that might shed light on the mechanisms by which PLOD3 contributes to the observed cellular phenotypes.

## 2. Results

### 2.1. The Expression and Prognostic Values of PLOD3 in Human Cancers

Our previous studies of human miR-210 knockout cells identified PLODs as potential miR-210 targets [42].miR-210 is a hypoxia-induced microRNA, and the link between PLOD3 and hypoxia in cancers has been reported [25,34]. Numerous studies have also revealed PLOD3 overexpression in human cancers (see **Introduction**). Our own, most updated analyses [43] confirmed that the PLOD3 mRNA level was indeed upregulated in most, although not all, tumors compared to the adjacent normal tissues (Supplementary Figure S1).

**Figure 1:**
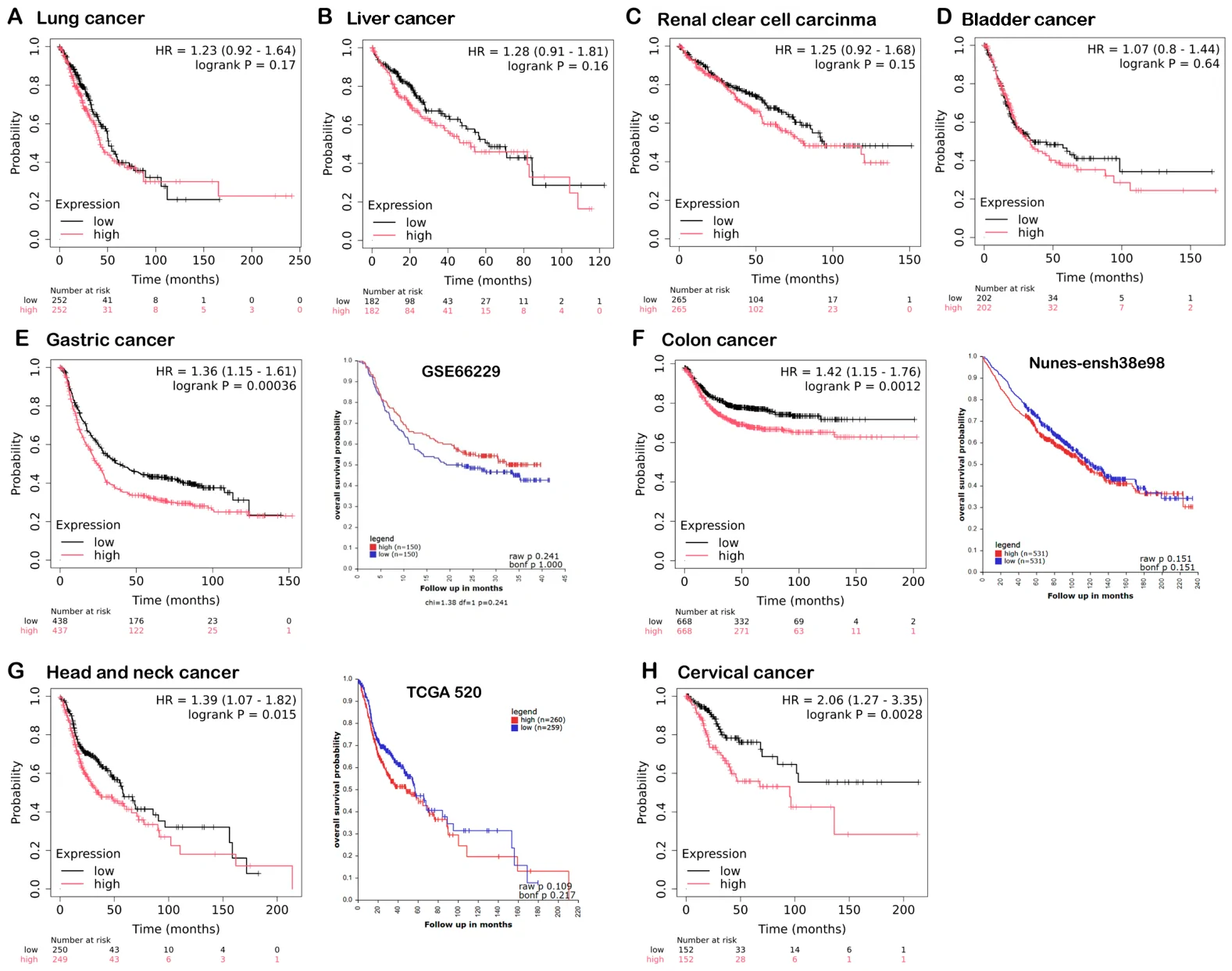
Relationship between PLOD3 mRNA expression and total survival probability in cancers. (**A**) Lung cancer, KM curve result from KMplotter. (**B**) Liver cancer, KM curve from KMplotter. (**C**) Kidney renal clear cell carcinoma, KM curve from KMplotter. (**D**) Bladder cancer, KM curve from KMplotter. (**E**) Gastric cancer, KM curve results from KMplotter (left panel) and R2 (right panel). For R2 the dataset used is indicated on the graph. (**F**) Colon cancer, KM curve results from KMplotter (left panel) and R2 (right panel). (**G**) Head and neck cancer, KM curve from KMplotter. (**H**) Cervical cancer, KM curve from KMplotter.

We then used three online KM plotting tools: GEPIA3 (https://gepia3.bioinfoliu.com) [43], KMplotter (https://www.kmplot.com/) [44], and R2 (https://hgserver1.amc.nl/), and to evaluate the relationship between PLOD3 mRNA and patient survival. These three sources use overlapping databases to calculate survival; GEPIA3 and KMplotter combine different datasets for the same cancer, while R2 analyzes single datasets. Patients with higher PLOD3 had been reported to fare worse in glioma, lung cancer, gastric cancer, liver cancer, colon cancer, renal cell carcinoma, bladder cancer, head and neck cancer, and cervical cancer [16,17,19,20,21,23,24,25,26,27,28,29,32,33,34,37,38,39,40]. Unexpectedly, we obtained much weaker association than earlier reports (Figure 1). For example, KMplotter identified no significant correlation (*p* < 0.05) between PLOD3 and the overall survival probability in lung cancer, liver cancer, renal cell carcinoma, and bladder cancer (Figure 1A–D), and the same conclusions were reached using R2 and GEPIA3 (examples see Supplementary Figure S2A,B, Supplementary Table S1). Although KMplotter found significant correlation in gastric cancer, colon cancer, and head and neck cancer, R2 and GEPIA3 yielded different results (Figure 1E–G, Supplementary Figure S2C, Supplementary Table S1). There are no glioma datasets at KMplotter and GEPIA3, but different glioma datasets at R2 gave conflicting results (Supplementary Figure S2D, Supplementary Table S1), with no significant correlation between PLOD3 expression and the overall survival for glioblastoma, the more aggressive type of glioma (Supplementary Figure S2E, Supplementary Table S1). The only confirmation came with cervical cancer (Figure 1H), but it appeared that the same, single dataset was used by all three websites. Curiously, PLOD3 mRNA is not significantly upregulated in cervical cancers (CESC, Supplementary Figure S1; [26]). We did not detect consistently significant correlation between PLOD3 and survival in other cancers, such as uterine, pancreatic, and ovarian cancers, either (Supplementary Figure S2F–H, Supplementary Table S1). Reasons behind these discrepancies with previous studies are explained in **Discussion**, but these results also indicated that PLOD3’s roles in cancer cells merited further investigation.

### 2.2. PLOD3 Knockdown and Overexpression

**Figure 2:**
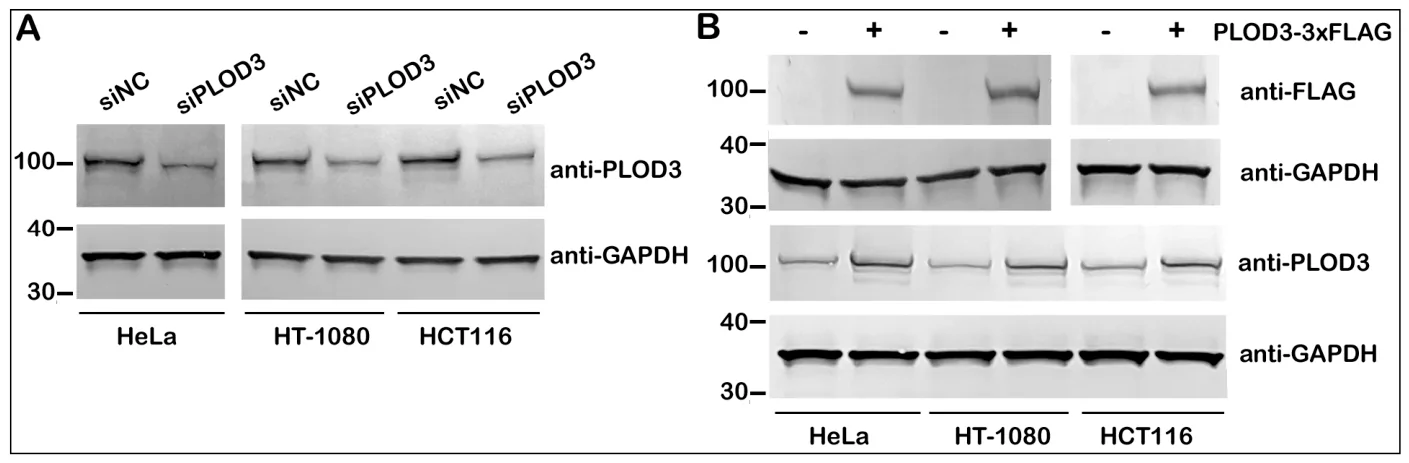
Western blot analyses of HeLa, HT-1080, and HCT116 cells undergoing RNAi against PLOD3 or PLOD3 overexpression. (**A**) Cells were transfected with the control siNC or siPLOD3 for 4 days. Cell extracts were run on a 4–20% SDS-PAGE gel, and blots were probed with antibodies against PLOD3 and the control GAPDH. Positions of the molecular weight markers are indicated on the left (in kilodaltons). (**B**) Cells were transfected with an empty vector or pCMV-PLOD3-3xFLAG for 2 days. Western blotting was performed to detect the FLAG-tagged protein, total PLOD3, and the control GAPDH, as described in (**A**). The endogenous PLOD3 and PLOD3-3xFLAG proteins were difficult to separate on the 4–20% SDS-gels, and since the PLOD3 and FLAG antibodies were both mouse antibodies, two blots with the same cell extract samples loaded were needed to detect PLOD3-3xFLAG and total PLOD3 separately, as shown here.

To study the functions of PLOD3, we used a small interfering RNA (siRNA) to knockdown PLOD3 expression and a PLOD3-3xFLAG encoding plasmid to overexpress PLOD3. For experimental models we employed three human cancer cell lines popular for biological and mechanistic studies: HeLa, HT-1080, and HCT116, representing cervical cancer, sarcoma, and colon cancers, respectively. Cervical cancer survival correlated with PLOD3 expression, albeit based on a single dataset (Figure 1H), a number of studies have looked at PLOD3 in colon cancer [18,19,20,21], and PLOD3 mRNA expression was elevated in sarcoma (SARC, Supplementary Figure S1). We first used an siRNA to transiently knockdown PLOD3 expression. Figure 2A shows that the endogenous PLOD3 protein expression was reduced by approximately 80% in all three cell lines four days after the PLOD3 siRNA (siPLOD3) transfection, compared to the control siRNA (siNC) transfection. We also overexpressed PLOD3 by transfecting cells with the pCMV-PLOD3-3xFLAG plasmid. Two days after transfection, we performed Western blotting and detected PLOD3-3xFLAG expression using the FLAG antibody (Figure 2B, upper panels). Probing with the PLOD3 antibody revealed that the degree of overexpression (OE) was no more than two times of that of the endogenous protein in the empty vector control transfection (Figure 2B, lower panels). Such modest overexpression could be due to the already high level of endogenous PLOD3, but it also likely avoided overwhelming the system with too much PLOD3 and inducing artifacts. In subsequent experiments we examined the phenotypes of PLOD3 knockdown cells 4–6 days after siRNA transfection or PLOD3 overexpressing cells 2–3 days after plasmid transfection.

### 2.3. The Effects of Decreased and Increased PLOD3 Expression on Cellular Phenotypes

**Figure 3:**
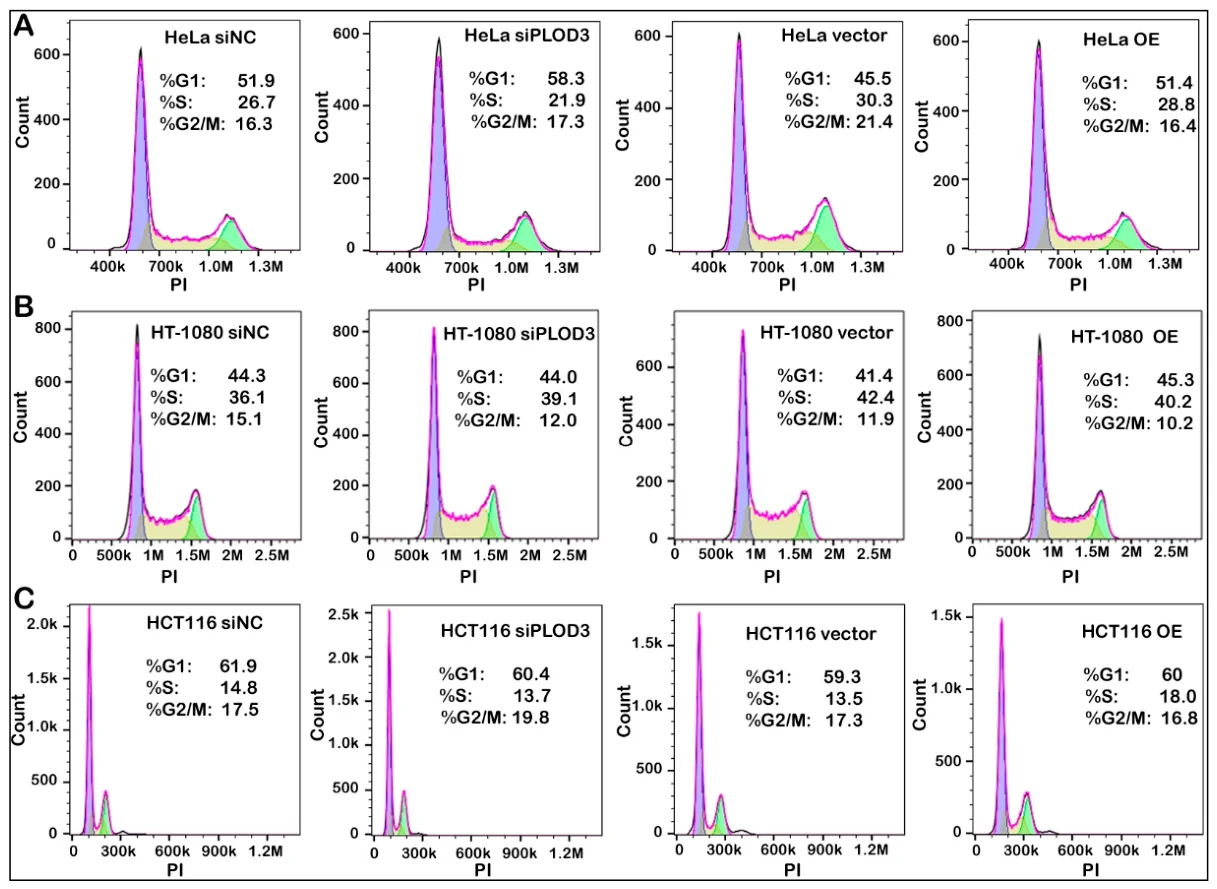
Representative cell cycle profiles of cells with different PLOD3 expression. The *y*-axis is the cell count, and *x*-axis propidium iodide (PI) staining for DNA content. The percentages of cells in the G1, S, and G2/M phases were determined automatically by the default settings of the cytometry software and listed inside the graphs. (**A**) HeLa cells, (**B**) HT-1080 cells, (**C**) HCT116 cells, panels from left to right were cells transfected with the control siNC, siPLOD3, the empty vector, and the pCMV-PLOD3-3xFLAG plasmid.

We first examined the cell cycle profiles by flow cytometry, and representative results are shown in Figure 3. The most consistent and significant change in HeLa cells was that siPLOD3 reduced the S phase cell population compared to the siNC treatment (Figure 3A). In contrast, siPLOD3 enhanced the S phase population in HT-1080 cells (Figure 3B). For HCT116 cells, PLOD3 OE increased the S phase population (Figure 3C). Thus, PLOD3 appeared to exert a similar trend of effects on the cell cycle of HeLa and HCT116 cells but the opposite effect on HT-1080 cells.

**Figure 4:**
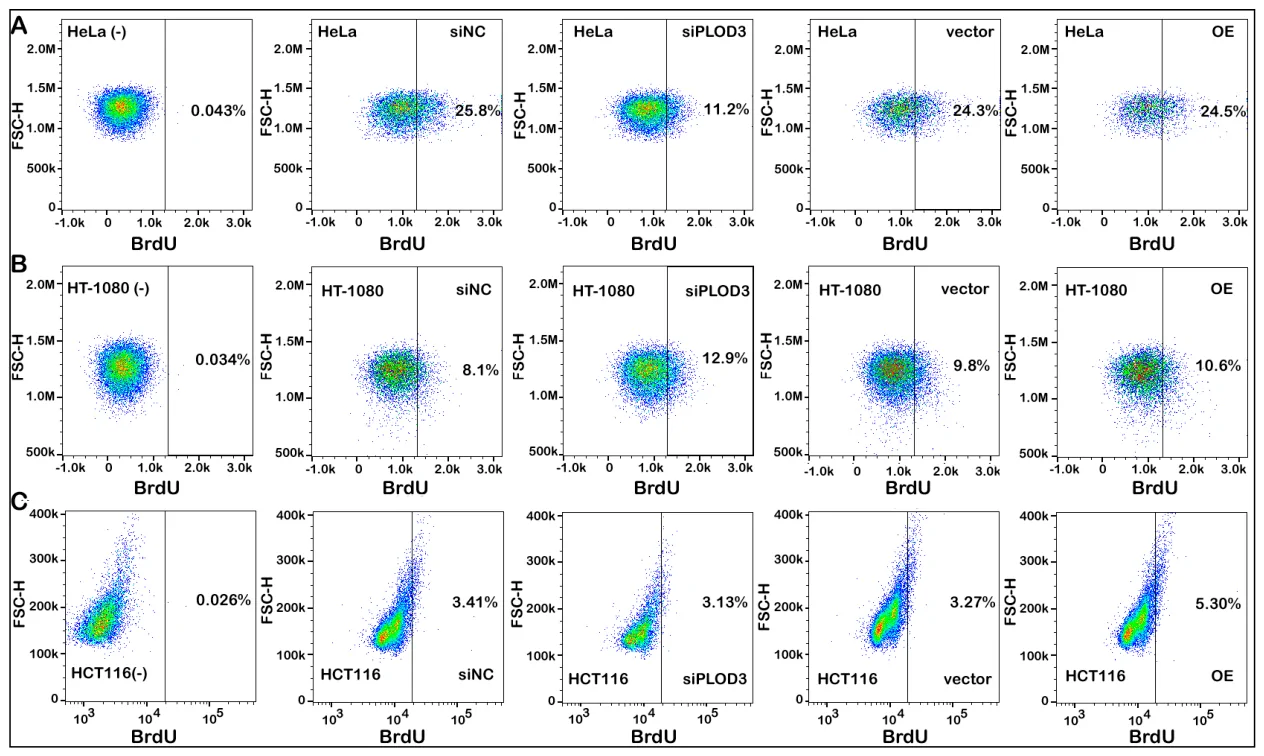
BrdU labeling of cells undergoing PLOD3 RNAi or OE.HeLa (**A**), HT-1080 (**B**), and HCT116 (**C**) cells were transfected with the control siNC, siPLOD3, an empty vector, or pCMV-PLOD3-3xFLAG.On the day of harvest, cells were first incubated briefly with BrdU before being processed for analyses. The *y*-axis is cell scattering, the *x*-axis is FITC signals indicative of BrdU incorporation. Representatives of three experiments are shown. The leftmost panels are cells not treated with BrdU, hence the negative staining controls for gating of BrdU positive cells in the right. Percentages of BrdU positive cells are given on the graphs.

To confirm the influences on the S phase, we performed pulse BrdU labeling experiments. HeLa cells transfected with the siPLOD3 had fewer BrdU-labeled cells than those transfected with the control siRNA (11.2% vs. 25.8%, Figure 4A). But HT-1080 cells transfected with siPLOD3 had 12.9% BrdU positive cells vs. siNC’s 8.1% (Figure 4B). PLOD3 OE increased BrdU-labeled HCT116 cells from 3.27% to 5.3% (Figure 4C). These BrdU results matched the cell cycle data, suggesting that PLOD3 produced variable effects on the cell cycle depending on the context of cancer cell lines.

**Figure 5:**
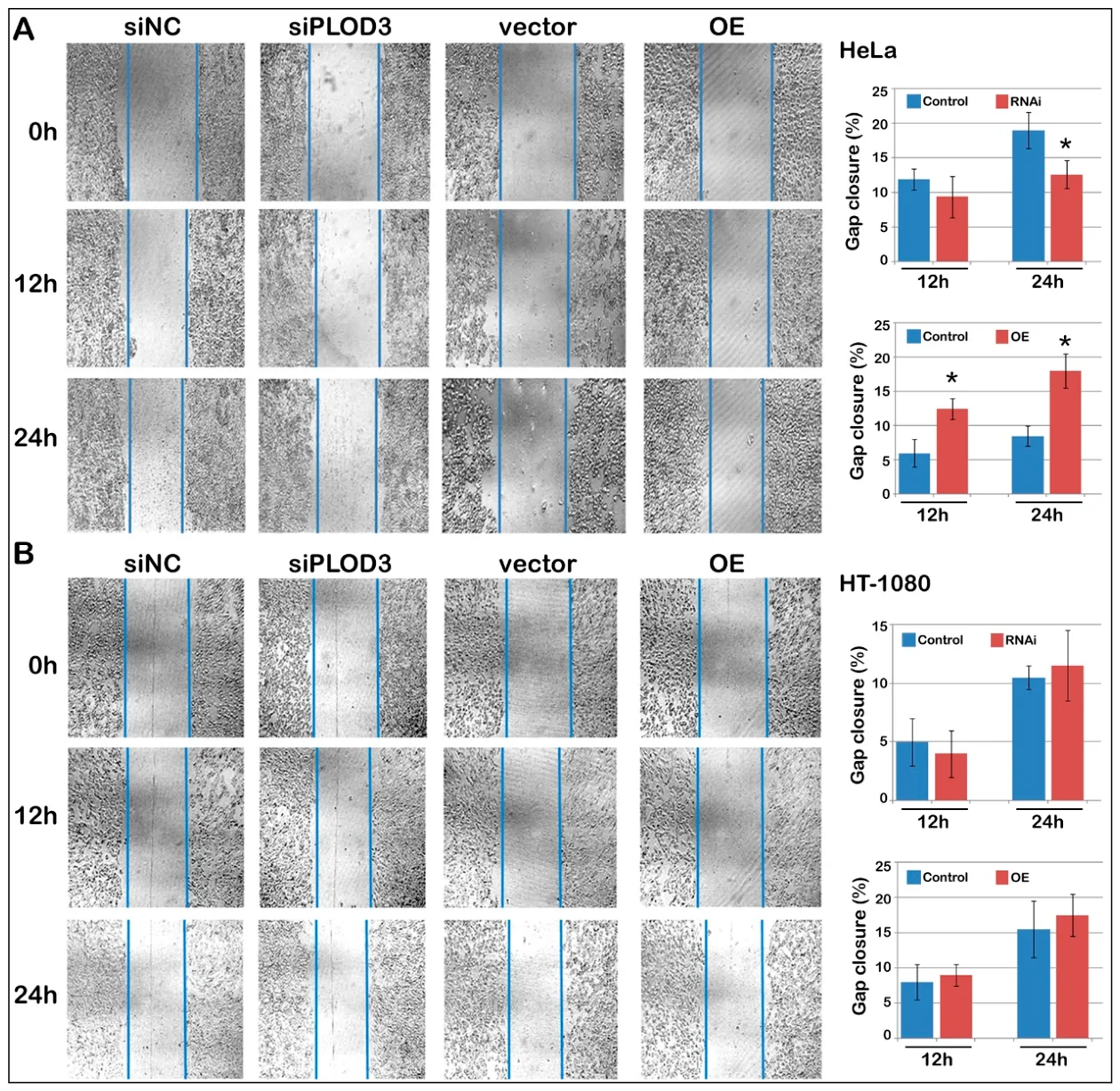
Scratch assays to measure cell migration. (**A**) Migration of HeLa cells undergoing PLOD3 RNAi or OE. Representative cell images at 0, 12, and 24 h are shown, with blue lines marking the gaps or leading edges of the cells. Gap closure (percentage) was calculated as the reduction in the gap width at 12 or 24 h from 0 h divided by the gap width at 0 h when the scratch was made. The right panels are the quantification for HeLa cells with PLOD3 RNAi and OE, with averages and standard deviations shown. Asterisks mark *p* < 0.05. (**B**) Migration of HT-1080 cells, descriptions as in (**A**).

Because PLOD3 modifies collagen, a major ECM component, we tested how altering PLOD3 affected cell migration using scratch assays. Figure 5A shows, with quantification graphs on the right, that siPLOD3 significantly decreased HeLa cell migration at 24 h, while PLOD3 OE significantly increased HeLa cell migration at both the 12 and 24 h time points. The migration of HT-1080 cells (Figure 5B) and HCT116 cells (Supplementary Figure S3), however, was unaffected by the status of PLOD3 expression.

**Figure 6:**
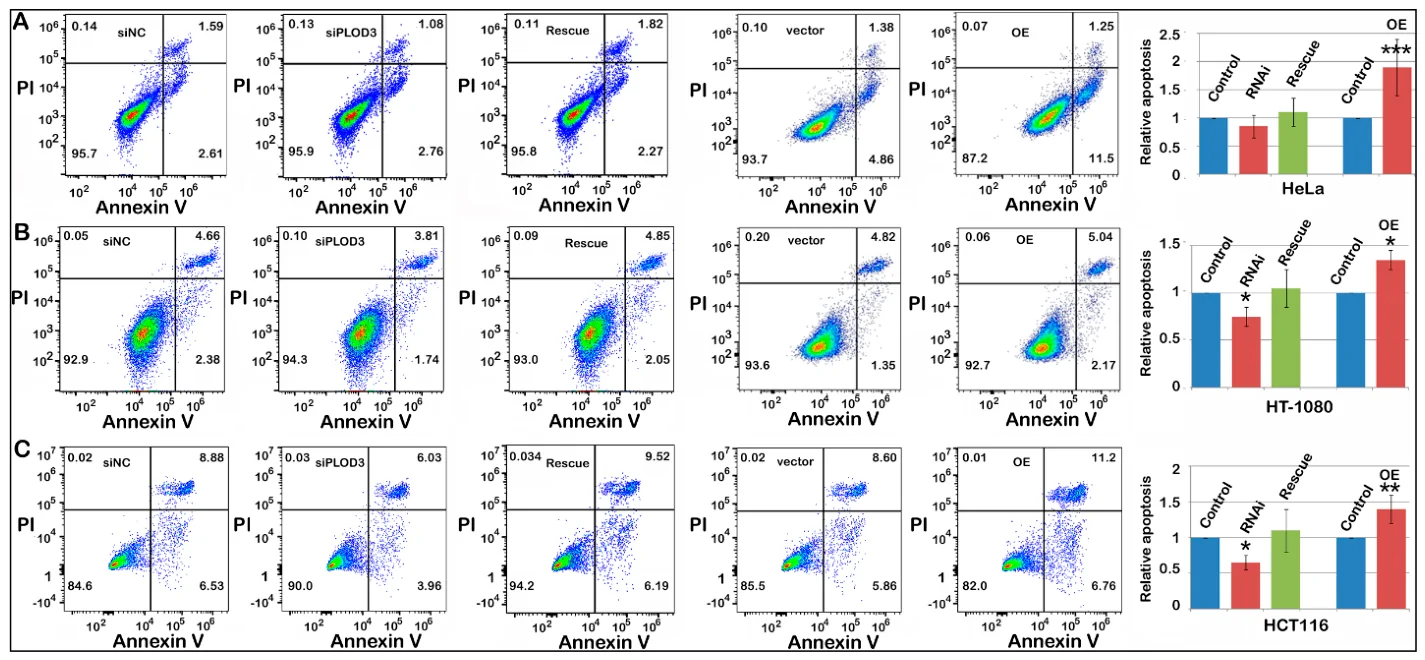
Apoptosis analyses. HeLa (**A**), HT-1080 (**B**), and HCT116 (**C**) cells were transfected with the control siNC, siPLOD3, siPLOD3+ the pCMV-PLOD3-3xFLAG plasmid (rescue), an empty vector, or the PLOD3-3xFLAG plasmid (OE), then subjected to PI and Annexin V staining and flow cytometry analyses. The *y*-axis, PI, detected DNA content. The *x*-axis, Annexin V, detected apoptosis, represented by the lower right quadrant and the upper right quadrant. Percentages of cells in each quadrant were determined automatically by the flow cytometry software and listed inside the graphs. The right panels present the quantifications, with averages and standard deviations of the percentages of total apoptotic cells. Compared to the control apoptosis arbitrarily set at 1, *: *p* < 0.05; **: *p* < 0.01; ***: *p* < 0.001.

Next we examined apoptosis in cells with different PLOD3 expression by Annexin V staining and flow cytometry. Annexin V marks apoptotic cells. We found that PLOD3 knockdown reduced the ratio of Annexin V positive cells—and, therefore, apoptosis—whereas PLOD3 OE enhanced apoptosis, in HeLa (Figure 6A), HT-1080 (Figure 6B), and HCT116 (Figure 6C). This indicated a more uniformed influence on apoptosis by PLOD3. To further confirm these results, we carried out rescue experiments by pCMV-PLOD3-3xFLAG plasmid transfection after siPLOD3 treatment. The plasmid transfection, in the continuous presence of siPLOD3, restored PLOD3 expression to approximately the endogenous level (Supplementary Figure S4), as well as raised apoptosis back to the control levels in the three cell lines (Figure 6A–C).

**Figure 7:**
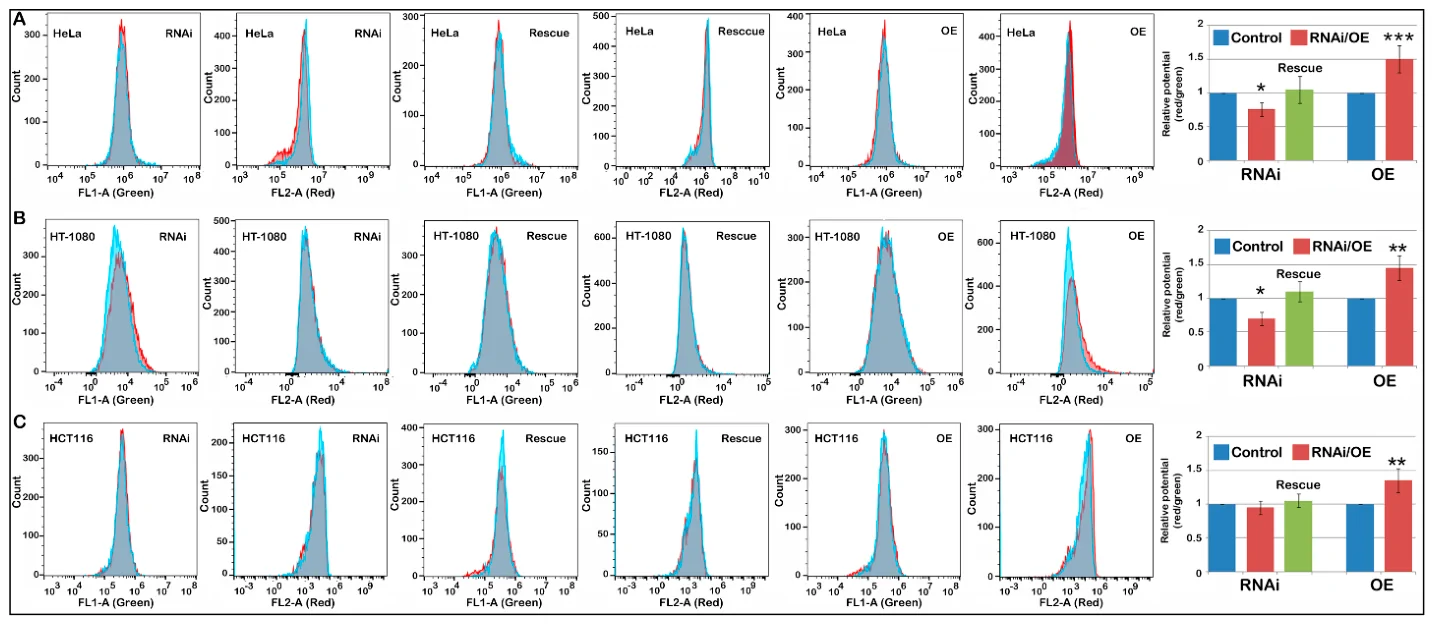
Relative mitochondrial membrane potentials of the cells with different PLOD3 expression. HeLa (**A**), HT-1080 (**B**), and HCT116 (**C**) cells were transfected with the control siNC, siPLOD3, siPLOD3+ pMCV-PLOD3-3xFLAG (rescue), control empty vector, or pCMV-PLOD3-3xFLAG (OE), then their mitochondrial membrane potentials examined by flow cytometry analyses. The *y*-axis is cell count, the *x*-axis green (FL1-A) or red (FL2-A) fluorescence signal. Green fluorescence signals were from the JC-1 monomers, and red from JC-1 aggregates. Blue peaks are signals from the control cells, overlaying red peaks of the RNAi, rescue, or OE cells. Right panels are the quantifications, showing the averages and standard deviations of the relative mitochondrial membrane potentials, calculated as the ratios of the red vs. green fluorescence signals. Comparing to the controls set at 1, *: *p* < 0.05; **: *p* < 0.01; ***: *p* < 0.001.

Many cellular phenotypes such as apoptosis are linked to the mitochondria. We thus measured the relative mitochondrial membrane potential in cells with different PLOD3 levels. Figure 7 shows results of the experiments. The blue peaks represent the control samples, and the red peaks the RNAi or OE samples; the FL1-A channel shows the green fluorescence emitted by the JC-1 dye monomers, formed under low membrane potential conditions, and the FL2-A channel shows the red fluorescence emitted by the JC-1 dye aggregates, formed under high membrane potential conditions. The ratio between the red and green fluorescence was then calculated to compare the relative mitochondrial membrane potentials between the control and RNAi/OE samples (Figure 7, right panels). We found that PLOD3 RNAi led to a modest but significant reduction (20–30%) in relative mitochondrial membrane potential in HeLa and HT-1080 cells, while PLOD3 OE elevated relative membrane potential by 30–50% in all three cell lines (Figure 7A–C). Again, ectopic PLOD3-3xFLAG expression rescued the siPLOD3-induced reduction of mitochondrial membrane potential (Figure 7).

**Figure 8:**
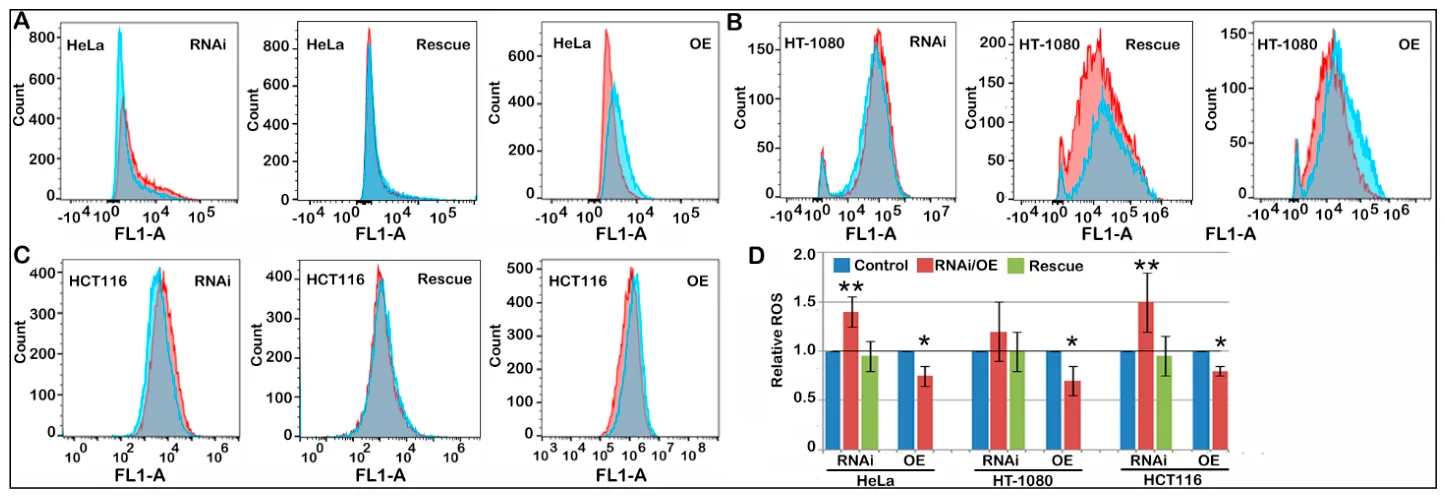
ROS analyses in cells undergoing PLOD3 RNAi and OE. HeLa (**A**), HT-1080 (**B**), and HCT116 (**C**) cells were transfected with the control siNC, siPLOD3, siPLOD3+ the PLOD3-3xFLAG plasmid (rescue), control empty vector, or the PLOD3-3xFLAG plasmid, then ROS examined by staining with the ROS-reactive dye, 2,7-Dichlorodi-hydrofluorescein diacetate, and flow cytometry. The *y*-axis is the cell counts, and the *x*-axis green (FL1-A) fluorescence signals by the dye. Blue peaks are signals of the control cells, overlaying red peaks of the RNAi, rescue, or OE cells. (**D**) The bar graph summarizes the quantification of relative ROS level (the *y*-axis), with that of the control cells set at 1. Averages and standard deviations of at least three biological replicates are shown. Comparing to the controls set at 1, *: *p* < 0.05; **: *p* < 0.01.

**Figure 9:**
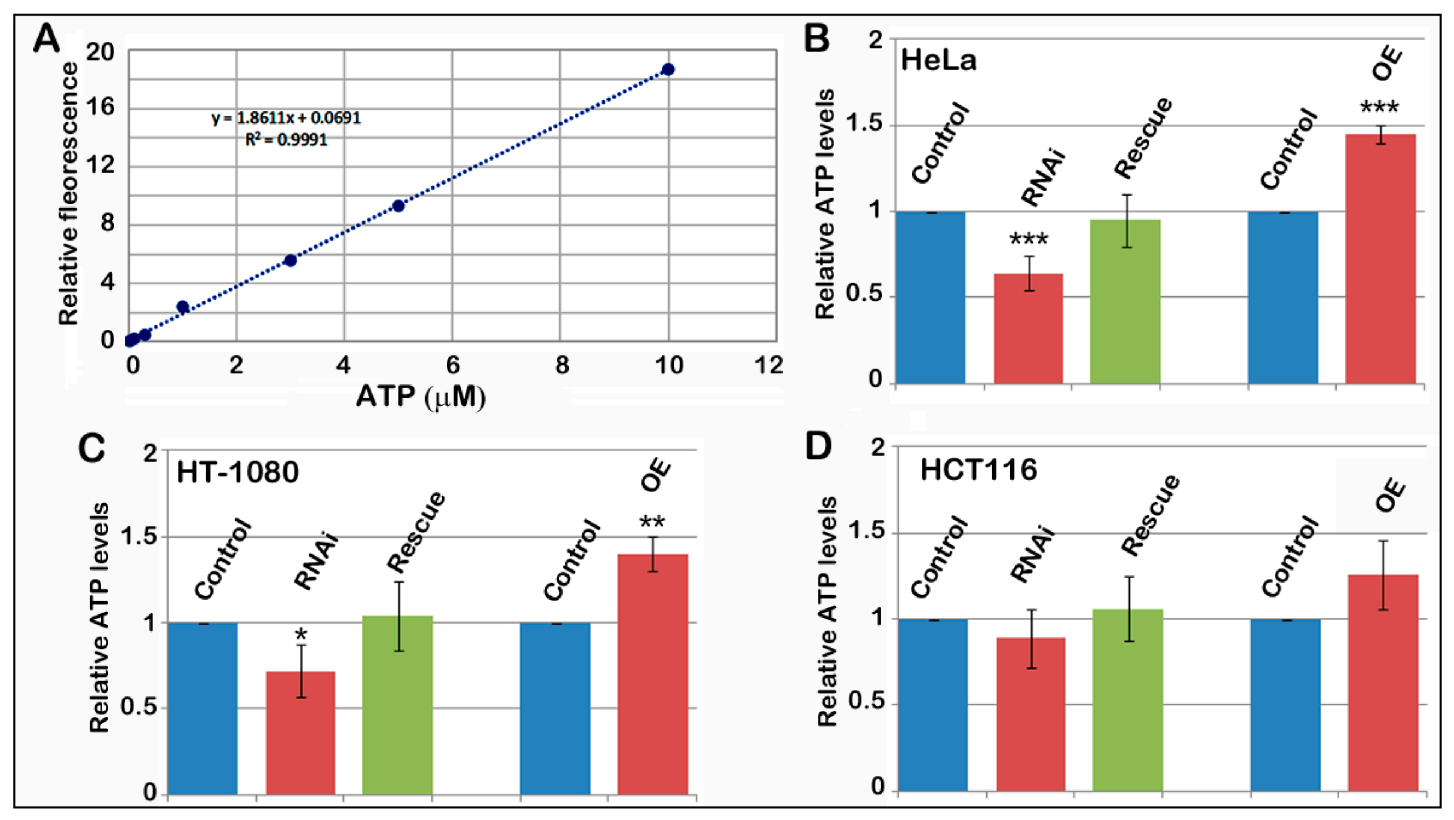
Quantification of ATP levels in cells. (**A**) An ATP standard curve assayed using a luminometer, showing the dynamic range within which our subsequent sample readings fell. Relative ATP levels (the *y*-axis) in HeLa (**B**), HT-1080 (**C**), and HCT116 cells (**D**), with that of the control cells set at 1. Averages and standard deviations are shown. Compared to the control cells, *: *p* < 0.05; **: *p* < 0.01; ***: *p* < 0.001.

We further measured the levels of ROS, a major product of mitochondrial metabolism, using 2,7-Dichlorodi-hydrofluorescein diacetate, which upon ROS oxidization emits green fluoresces and is detected on the FL1-A channel. Figure 8A–C show the overlays of blue (control) and red (RNAi/rescue/OE) peaks in the three cell lines, and Figure 8D the quantification. The overall trend was consistent among the cells: ROS levels were elevated by RNAi against PLOD3, but decreased by PLOD3 overexpression. Additional PLOD3-3xFLAG expression suppressed the siPLOD3-induced ROS increase, demonstrating the specificity of our results (Figure 8). We also quantified the ATP levels in cells. Figure 9A shows the linear range of our ATP assay within which our cellular readings fell. PLOD3 knockdown significantly reduced ATP levels in both the HeLa and HT-1080 cells, and PLOD3-3xFLAG expression rescued or enhanced the ATP levels (Figure 9B,C). ATP change in HCT116 was not statistically significant, but the trend was nevertheless consistent with those of the HeLa and HT-1080 cells (Figure 9D).

### 2.4. The Effects of PLOD3 Perturbation on Global Gene Expression

**Figure 10:**
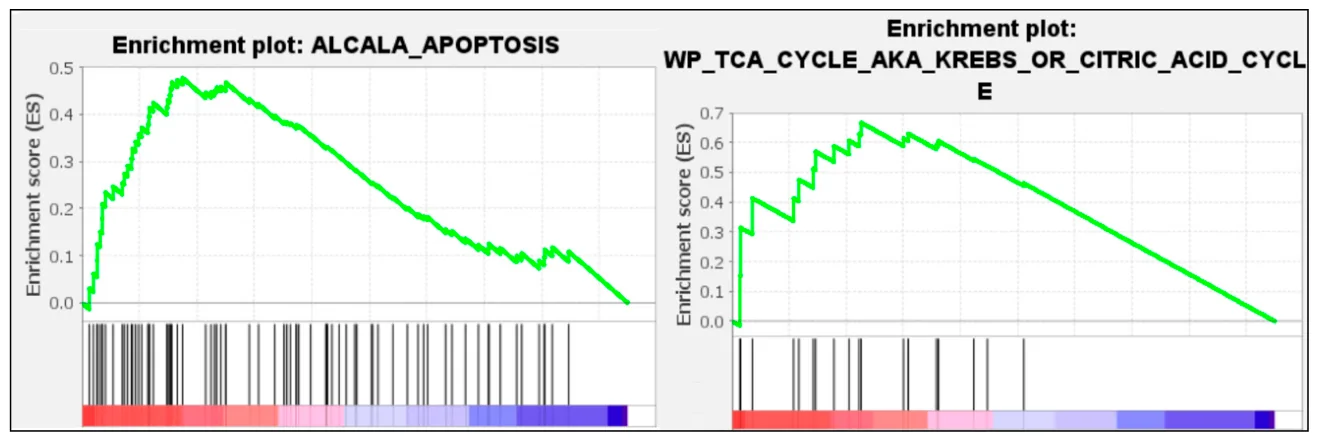
Expression of gene sets enriched by PLOD3 OE according to GSEA. Normalized Enrichment Score= 1.96 (apoptosis) or 1.95 (Krebs cycle).

To elucidate the mechanisms by which PLOD3 regulates the phenotypes of cancer cells, we performed RNA-seq on HeLa, HT-1080, and HCT116 cells with different PLOD3 levels. We then carried out Gene Set Enrichment Analysis (GSEA) [45] comparing RNA-seq data across all three cell lines. As shown in Figure 10, PLOD3 OE altered the expression of the apoptosis gene set and the Krebs cycle gene sets. These results supported the observations that PLOD3 OE enhanced apoptosis as well as modulated the mitochondrial metabolism in HeLa, HT-1080, and HCT116 cells (Figure 6, Figure 7, Figure 8 and Figure 9).

We also derived the differentially expressed genes and subjected them to functional, GO and pathway analyses [46]. Supplementary Figure S5 shows that the top GO Molecular Function terms in both the RNAi and OE comparisons were tied to PLOD3’s enzymatic activity in collagen modification, consistent with PLOD3 expression changes. Table 1 then shows the significant terms in the UP_KW_BIOLOGICAL_PROCESS category. The RNAi group contained the cell cycle and apoptosis terms, and the OE group contained the electron transport and respiratory chain terms.

Table 2 shows the top terms in the GOTERM_BP_DIRECT category. Both the RNAi and OE groups had terms related to apoptosis process, and OE had a number of terms associated with mitochondrial functions, such as “proton motive force-driven mitochondrial ATP synthesis”, “aerobic respiration”, “mitochondrial electron transport”, and “response to oxidative stress”. Table 3 shows the top terms in the KEGG_PATHWAY category. The RNAi group and OE group contained the metabolic pathway terms, and the latter further had the ROS and oxidative phosphorylation terms. Previous studies had shown that PLOD3 induced TWIST1 expression or the NEDD4/GOT2 axis to impact renal cell carcinoma [31,34], regulated the expression of YAP1 in lung cancer [29], or enhanced TM9SF4-mediated autophagy in colon cancer [21]. These genes were not identified as differentially expressed in our RNA-seq dataset (GSE343753). But our GSEA, GO, and pathway analyses confirmed that PLOD3 could alter gene expression linked to the cellular responses to apoptosis and mitochondrial metabolism, providing potential mechanisms to the phenotypes observed above.

## 3. Discussion

In this study we first revisited the relationship between PLOD3 mRNA expression and human cancer survival and found that high PLOD3 did not consistently or significantly correlate with worse survival in most cancers. We then investigated how altering PLOD3 levels by RNAi and overexpression impacted the phenotypes of HeLa, HT-1080, and HCT116 cells, three representative human cancer cell lines. PLOD3 expression had a variable effect on the cell cycle and cell migration behaviors in the three cell lines. However, increased PLOD3 induced apoptosis, elevated the mitochondrial membrane potential and ATP levels, and reduced the ROS levels in all three cell lines. Subsequently, RNA-seq analyses revealed changes in biological processes and pathways that might provide the mechanisms underlying these phenotypes.

Previous studies had shown that PLOD3 expression was increased in human tumor samples compared to the adjacent normal tissues, and that higher PLOD3 was associated with poorer survival in several cancers (see **Introduction**). We confirmed increased PLOD3 mRNA levels in a majority of human tumors (Supplementary Figure S1), but also demonstrated that higher PLOD3 did not significantly foretell worse patient outcomes (Figure 1, Supplementary Figure S2, Supplementary Table S1). There may be two reasons for the conflicting results. One is that we used the most up-to-date data on three different websites (https://www.kmplot.com/, https://hgserver1.amc.nl/, and https://gepia3.bioinfoliu.com) to evaluate and compare the KM plots, while previous work examined a smaller patient population. The other is that most studies let the online tools gate PLOD3 expression automatically, which often resulted in vastly uneven numbers of patients in the low and high PLOD3 groups and skewed more favorable prognostic *p* values. In contrast, we always divided the patient populations by the median PLOD3 expression and, therefore, set a strict and unbiased criterion for all the datasets and cancers. Curiously, cervical cancer is the only cancer that shows a strong prognostic association with PLOD3 (Figure 1), despite PLOD3 mRNA not being overexpressed in cervical cancer (Supplementary Figure S1). This unique conclusion might arise from the fact that all three online tools carried the same, single clinical cervical cancer dataset, with fewer patients than other cancer types. More cervical cancer patient data could help clarify the situation. A limitation of our study is that we looked at the effects of only one gene, PLOD3, not assessing the likelihood that PLOD3 works along with other genes to impact prognosis. PLOD3 acts on collagen and ECM.ECM impacts the survival, migration, and metastasis of cancer cells, although the contribution by ECM to tumorigenesis is multifaceted and complex [3,4], so naturally PLOD3 may not have a simple or straightforward role in cancer. PLOD3 might exert divergent effects depending on the cancer types, and more patient data will help ascertain the relationship between PLOD3 expression and cancer prognosis.

Another outstanding question is how PLOD3 controls cancers. Perhaps due to the prevailing notion that higher PLOD3 equated with worse outcomes, earlier studies had aimed for the role of PLOD3 as an oncogene in cell lines of single cancer types, but there had been little overlap in the reported PLOD3 mechanisms in different cancers, suggesting that PLOD3 might exert pleiotropic and cell-specific effects. So our work examined three cell lines, HeLa, HT-1080, and HCT116, derived from three different human cancers, performed the same assays to allow for side-by-side, systemic analyses of PLOD3’s roles in human cancer cells. We used RNAi to knockdown PLOD3 and a PLOD3-3xFLAG plasmid to overexpress PLOD3 (Figure 2), and compared if the treatments acted oppositely on cellular phenotypes. In terms of the cell cycle, PLOD3 had a stimulating effect on the S phase in HeLa and HCT116, but an opposing effect in HT-1080 (Figure 3), and this finding was supported by the BrdU labeling experiments (Figure 4). PLOD3 also facilitated migration of HeLa cells, but had no effect on HT-1080 and HCT116 cells (Figure 5, Supplementary Figure S3). Thus, different cells varied in their requirements for PLOD3 levels, revealing the nuanced nature of the regulation by PLOD3. There were, however, also phenotypes common among the three cell lines. RNAi knockdown of PLOD3 reduced apoptosis, the mitochondrial membrane potential, and ATP levels, but increased ROS, while PLOD3 overexpression had the opposite effects (Figure 6, Figure 7, Figure 8 and Figure 9). Taken together, we conclude that PLOD3 has broad, context-dependent, partially conserved functions across human cancer cell lines.

A new feature of our work is that we further carried out transcriptomics analyses to support the observed phenotypes and gain mechanistic insights. Figure 10 shows cells had gene sets altered in the apoptosis and Krebs cycle processes, supporting the observed PLOD3 phenotypes (Figure 6, Figure 7, Figure 8 and Figure 9). The apoptotic process and the metabolic and oxidative pathways were also identified by GO and pathway analyses (Table 1, Table 2 and Table 3), suggesting that the functional effects of PLOD3 might, directly or indirectly, encompass these two major processes: apoptosis and mitochondrial function. Future studies will verify the expression of important genes and examine their roles in those specific biological processes.

Considering the previously reported PLOD3 phenotypes, Tsai et al. [25] showed that siPLOD3 reduced BrdU incorporation in glioma cells, which was broadly in line with our observation in HeLa and HCT116 cells (Figure 4). Our results indicated PLOD3 facilitated apoptosis (Figure 6), but Baek et al. [41] showed siPLOD3 increased apoptosis in lung cancer cells, although Ding et al. [40] reported no apoptotic effect by PLOD3 in colon cancer cells. We found PLOD3 had a positive effect on migration in HeLa cells (Figure 5), consistent with reports that siPLOD3 suppressed cell migration in cell lines from lung cancer [27], kidney renal clear cell carcinoma [31,34], gastric cancer [17], colon cancer [21,40], head and neck cancer [38], and liver cancer [24]. Taken together, our results have both agreements and disagreements with the literature. We examined and compared diverse cell lines in the same study, and the concurrent RNA-seq experiments identified changes associated with the phenotypes, yet discrepancy with published reports might still arise from the varying times of siRNA treatments, the different cell lines and experimental systems employed, and the different assays performed. The mRNA levels of collagens were not significantly altered by our PLOD3 manipulation (GSE343753), and our two-dimensional in vitro cell culture models could afford only limited tests of ECM.An additional limitation of this study is that we treated cell lines with siRNAs for only up to 6 days and the PLOD3 OE lasted for only up to 3 days, yet the long term effects of PLOD3 as well as its in vivo functions could be different. Thus, more work is needed to establish the functions and mechanisms of PLOD3.

An interesting finding here is that PLOD3 RNAi and OE altered the mitochondrial membrane potential, ROS, and ATP levels (Figure 7, Figure 8 and Figure 9), which was supported by RNA-seq experiments and analyses (Figure 10, Table 1, Table 2 and Table 3). This phenotype had not been reported for human PLOD3, although a recent study demonstrated that zebrafish PLOD3 deficiency downregulated mitochondrial metabolism [47]. The relationship between metabolism and cancers is well known, and mechanical stress, often mediated through ECM, influences cellular metabolism [48]. We note that bioinformatics analyses identified terms about rRNA, ribosome, and translation (Table 1, Table 2 and Table 3), which might also signal metabolic changes. Hence our data suggested a new mechanism by which PLOD3 might contribute to cellular regulation and potentially carcinogenesis, and more work is needed to validate the finding and elucidate how PLOD3 functions might intersect with mitochondrial metabolism. In summary, our study unveiled a complex relationship between PLOD3 expression and cancer prognosis, identified the broad, sometimes variable impacts by PLOD3 on critical cellular processes in human cancer cells, and provided a framework for investigating PLOD3 contribution to cancers and the underlying mechanisms in the future.

## 4. Materials and Methods

### 4.1. PLOD3 Expression and KM Plot Analyses

The mRNA expression of PLOD3 in various tumors and the adjacent normal tissues was examined with the GEPIA3 tool (https://gepia3.bioinfoliu.com, last accessed on 22 August 2026). For KM plotting of the relationship between PLOD3 mRNA levels and cancer patient survival, data were evaluated on three websites: https://www.kmplot.com/, https://hgserver1.amc.nl/, and https://gepia3.bioinfoliu.com, last accessed on 25 August 2026. Patient cohorts were divided by their median PLOD3 expression.

### 4.2. Plasmid Construction and siRNAs

The coding sequence of the human full length PLOD3 (NM_001084.5) was synthesized and cloned into the p3xFLAG-CMV10 vector to produce pCMV-PLOD3-3xFLAG by General Biology, Hefei, China. This plasmid expresses the PLOD3 protein tagged at its C-terminus with the 3xFLAG epitope. The endogenous PLOD3 gene encodes a signal peptide in its N-terminus that enables PLOD3’s insertion into the membrane of the endoplasmic reticulum. To avoid interfering with this signal peptide function, we therefore appended the 3xFLAG tag to the C-terminus of PLOD3. A control siRNA and a PLOD3 siRNA were ordered from and synthesized by Sangon (Shanghai, China). The PLOD3 siRNA (5′-GGAAGUACAAGGAUGAUGACG-3′, nucleotides 855–875 in NM_001084.5) was based on Baek et al. (2018) [27].

### 4.3. Cell Cultures

The HeLa, HT-1080, and HCT116 cell lines were acquired from BD Bio (Hangzhou, Zhejiang, China). HeLa cells were cultured in Dulbecco’s modified Eagle’s medium supplemented with 10% fetal bovine serum and 2 mM L-glutamine (Invitrogen, Waltham, MA, USA). HT-1080 cells were cultured in the Eagle’s minimum essential medium supplemented with 10% fetal bovine serum and 2 mM L-glutamine. HCT116 cells were cultured in the McCoy 5A medium supplemented with 10% fetal bovine serum and 2 mM L-glutamine. All cells were maintained at 37 °C with 5% CO_2_ in a cell culture incubator. Cells were transfected with plasmids or siRNAs, with approximately 400 ng of total nucleic acids per well in a 24-well plate, using Lipofectamine 2000 (Invitrogen). For rescue experiments, cells were transfected with siRNAs for 4 days, transferred to a 6-well plate, then transfected with 2000 ng of an empty vector or pCMV-PLOD3-3xFLAG for 2 days before analyses.

### 4.4. Protein Analyses

Cell lysis, protein extraction, Western blotting, and image acquisition and quantification were performed as described [42]. The primary FLAG antibody was from Sigma (dilution 1:5000, catalog number F3165, St Louis, MO, USA), GAPDH antibody from Solarbio (dilution 1:2000, catalog number K200057M, Beijing, China), and PLOD3 antibody from Proteintech (dilution 1:2000, catalog number 60058-1-Ig, Rosemont, IL, USA).

### 4.5. Cell Phenotype Analyses

The cell cycle profiles were examined using the Cell Cycle and Apoptosis Analysis Kit (YEASEN, Shanghai, China) [42]. Cells were transfected with an siRNA or plasmid DNA in a 24-well plate and, when confluent, transferred to a 6-well plate. Foursix days after siRNA transfection or two-three days after plasmid transfection, cells were washed, trypsinized, and collected by centrifugation. Cells were fixed in cold 70% ethanol and then stained with a PI solution containing RNase A. Stained cells were analyzed using a BD Accuri C6 flow cytometer and the FlowJo v10.6 software (Becton Dickinson, Franklin Lakes, NJ, USA). Cells at different phases of the cell cycle were determined automatically using FlowJo default settings.

BrdU labeling experiments were carried out to measure cells in the S phase using the FITC-BrdU Cell Proliferation Detection Kit (KeyGenBioTech, Nanjing, China). Cells were transfected with the siRNAs or plasmids, and 4–6 days (siRNA) or 2–3 days (plasmid) later, labeled with BrdU (Sigma) at 10 μM for 60 min. Cells were harvested, DNA denatured, and incubated with a FITC-BrdU antibody according to the KeyGenBioTech instructions. Cells were then analyzed by flow cytometry as described above.

For scratch assays to measure migration [49], transfected cells seeded in a 12-well plate were grown to 80% confluence, then a pipet tip used to make a scratch across the cell monolayer surface. Floating cells were removed, and the remaining cells were maintained in serum-free media for the next 24 h. At 0, 12, and 24 h the scratch or the gap it introduced was monitored under a light microscope, pictures taken, with cells entering the 0 h gap area considered migration.

Apoptosis was assessed using the Annexin V-Alexa Fluor 647/PI Apoptosis Detection Kit (YEASEN) [42]. Cells were transfected with siRNAs for 4–6 days or plasmids for 2–3 days. Cells were harvested, stained with PI and Annexin V-Alexa Fluor 647, and then analyzed using a BD Accuri C6 flow cytometer and the FlowJo v10.6 software as described above.

The mitochondrial membrane potential was measured using the JC-1 Mitochondrial Membrane Potential Assay from Sciben (Nanjing, China) [42]. The JC-1 dye accumulates in the mitochondria. When the membrane potential is low, JC-1 exists as a monomer which emits green fluorescence. When the membrane potential is high, JC-1 aggregates to yield red fluorescence. The ratio of red vs. green fluorescence thus serve as an index of the mitochondrial membrane potential. siRNA or plasmid-transfected cells were stained with JC-1, washed, and then analyzed using the BD cytometer. The FlowJo v10.6 software was used to quantify red and green fluorescence signals.

ROS levels were measured using the Meilun Reactive Oxygen Species Assay Kit (Meilunbio, Dalian, China) [42], which is based on 2,7-Dichlorodi-hydrofluorescein diacetate, a dye that fluoresces upon ROS oxidization. Cells transfected with siRNA or plasmid were stained with the dye for flow cytometry studies, and cells were excited at 488 nm and detected at 525 nm. Fluorescence signals were quantified using the FlowJo v10.6 software.

ATP levels were determined using the ATP Assay Kit from Beyotime (Shanghai, China). Cells were lyzed in the extraction buffer provided by the kit, and a normalized amount of lysates/proteins mixed with the ATP assay substrates, and signals acquired using a luminometer, according to the manufacture’s instruction.

All experiments had at least three biological replicates.

### 4.6. RNA-Seq and Data Analyses

Total RNA was isolated from the siRNA or plasmid transfected HeLa, HT-1080, and HCT116 cells using Trizol (Invitrogen). For RNA-seq (General Biology), 1 μg of total RNA was used to prepare a library. Poly(A) mRNA was enriched using the oligo(dT) beads. mRNA was fragmented by divalent cations at high temperature. cDNA was synthesized following priming with random primers. Double-stranded cDNA was purified, ends filled, a dA-tail added, followed by T-A ligation to introduce adaptors. Adaptor-ligated DNA was selected by size using DNA Clean Beads and then amplified by PCR. Finally, libraries were multiplexed and loaded onto an Illumina HiSeq/Illumina Novaseq/MGI2000 instrument for sequencing using a 2 × 150 paired-end (PE) configuration according to the manufacturer’s instructions (Illumina, San Diego, CA, USA).

Raw sequencing reads in FASTQ format were treated and cleaned up with Fastp (v0.24.1). Remaining sequences were processed by Cutadapt (V1.9.1), and data were aligned to the human reference genome GRCh38 with HISAT2 (v2.2.1). Gene-level counts were quantified using HTSeq (v0.6.1). RNA-seq data have been deposited in the Gene Expression Omnibus under the accession number GSE343753. Student’s t test was performed to identify differentially expressed genes (*p* < 0.05). GSEA [45] and DAVID (https://davidbioinformatics.nih.gov) [46] were used to analyze differences in gene set enrichments as well as GO (Gene Ontology) terms and KEGG (Kyoto Encyclopedia of Genes and Genomes) pathways, last accessed on 25 August 2026.

### 4.7. Statistical Analysis

GraphPad Prism 7.0 (GraphPad Software, San Diego, CA, USA) was used to perform the two-sided, two-sample unequal variance Student’s t-test and Dunnett’s test, with *p* value < 0.05 considered statistically significant.

## Figures and Tables

**Table 1:** Terms in the UP_KW_BIOLOGICAL_PROCESS category (*p* < 0.05) for the differentially expressed genes in the PLOD3 RNAi or OE cells, compared to their respective control cells.

UP_KW_BIOLOGICAL_PROCESS			
RNAi	*p* Value	OE	*p* Value
rRNA processing	9.8 × 10^−4^	Electron transport	2.2 × 10^−4^
Protein transport	6.8 × 10^−3^	Respiratory chain	2.4 × 10^−4^
Cell cycle	8.5 × 10^−3^	Host–virus interaction	7.5 × 10^−4^
Host–virus interaction	1.5 × 10^−3^	Hydrogen ion transport	3.9 × 10^−3^
Ribosome biogenesis	0.017	Antiviral defense	0.014
Autophagy	0.017	rRNA processing	0.023
Apoptosis	0.019		
Cell division	0.037		

**Table 2:** Top terms in the GOTERM_BP_DIRECT category for the differentially expressed genes in the PLOD3 RNAi or OE cells, compared to the respective control cells.

GOTERM_BP_DIRECT	RNAi	*p* Value
rRNAprocessing		1.3 × 10^−4^
protein transport		1.6 × 10^−4^
phosphatidylinositol phosphate biosynthetic process		2.9 × 10^−4^
phosphatidylinositol-3-phosphate biosynthetic process		3.1 × 10^−4^
Endoplasmicreticulum unfolded protein response		7.2 × 10^−4^
apoptotic process		8.0 × 10^−4^
regulation of autophagy		1.0 × 10^−3^
commissural neuron axon guidance		1.7 × 10^−3^
cell migration		2.7 × 10^−3^
negative regulation of extrinsic apoptotic signaling pathway		6.5 × 10^−3^
**GOTERM_BP_DIRECT**	**OE**	***p* Value**
proton motive force-driven mitochondrial ATP synthesis		1.3 × 10^−8^
response to oxidative stress		9.7 × 10^−6^
translation		1.5 × 10^−5^
proton transmembrane transport		8.4 × 10^−5^
cellular response to oxidative stress		1.5 × 10^−4^
aerobic respiration		3.9 × 10^−4^
mitochondrial electron transport		1.9 × 10^−3^
rRNA processing		4.2 × 10^−3^
regulation of G1/S transition of mitotic cell cycle		7.4 × 10^−3^
apoptotic process		8.1 × 10^−3^

**Table 3:** Top terms in the KEGG_PATHWAY category for the differentially expressed genes in the PLOD3 RNAi or OE cells, compared to the respective control cells.

KEGG_PATHWAY	RNAi	*p* Value
Metabolic pathways		1.4 × 10^−4^
Autophagy—animal		2.8 × 10^−4^
Inositol phosphate metabolism		1.6 × 10^−3^
Lysosome biogenesis		2.1 × 10^−3^
Phosphatidylinositol signaling system		2.8 × 10^−3^
Salmonella infection		4.3 × 10^−3^
Prostate cancer		5.1 × 10^−3^
Tight junction		5.6 × 10^−3^
Bladder cancer		7.1 × 10^−3^
N-Glycan biosynthesis		9.0 × 10^−3^
**KEGG_PATHWAY**	**OE**	***p* Value**
Parkinson disease		7.4 × 10^−10^
Prion disease		6.6 × 10^−9^
Chemical carcinogenesis—reactive oxygen species		2.0 × 10^−7^
Oxidative phosphorylation		7.4 × 10^−7^
Amyotrophic lateral sclerosis		1.1 × 10^−6^
Pathways of neurodegeneration—multiple diseases		2.5 × 10^−6^
Ribosome		6.4 × 10^−5^
Alzheimer disease		1.0 × 10^−4^
Metabolic pathways		0.033
Necroptosis		0.037

## Data Availability

RNA-seq data generated in this work have been deposited in the Gene Expression Omnibus under the accession number GSE343753.

## Supplementary Materials

The following supporting information can be downloaded at: https://www.mdpi.com/article/10.3390/ijms27209336/s1.
